# Satisfaction of Basic Psychological Needs Leads to Better Academic Performance via Increased Psychological Capital: A Three-Wave Longitudinal Study Among High School Students

**DOI:** 10.3389/fpsyg.2019.02113

**Published:** 2019-09-18

**Authors:** Marcos Carmona-Halty, Wilmar B. Schaufeli, Susana Llorens, Marisa Salanova

**Affiliations:** ^1^Escuela de Psicología y Filosofía, Universidad de Tarapacá, Arica, Chile; ^2^Research Unit of Occupational & Organisational Psychology and Professional Learning, KU Leuven, Leuven, Belgium; ^3^Department of Social, Health & Organizational Psychology, Utrecht University, Netherlands; ^4^WANT Research Team, Universitat Jaume I, Castellón de la Plana, Spain

**Keywords:** basic psychological needs, academic PsyCap, academic performance, GPA, high school students

## Abstract

This brief report proposes a model in which academic psychological capital (PsyCap) mediates between the satisfaction of student’s basic psychological needs and their academic performance, as assessed by students’ GPA. Participants were 407 adolescents, aged 12–18, recruited from three Chilean schools. Through structural equation modeling, direct and indirect effects were calculated. Results show that academic PsyCap (assessed at time 2) fully mediates the relationship between the satisfaction of basic psychological needs (assessed at time 1) and academic performance (assessed at time 3). This means that students whose basic psychological needs are satisfied at school experience more hope, efficacy, resilience, and optimism (PsyCap), which, in turn, leads to better academic performance. Both theoretical and practical implications of the results are addressed, as well as strengths and weaknesses and directions for future research.

## Introduction

The emergence of positive education—focused on both traditional skills and happiness (Seligman et al., 2009)— has made it possible to incorporate some recent constructs embraced in positive psychology into the educational research agenda. One of them is academic PsyCap: a psychological state of development characterized by hope, efficacy, resilience, and optimism (Luthans et al., 2012). However, given the relative novelty of the PsyCap construct, little is known about how students’ perceptions of their school environment may predict its subsequent appearance (Carmona–Halty et al., 2018). The current study addresses this issue by proposing that the satisfaction of students’ basic psychological needs (BPN) –grounded in Self–Determination Theory (SDT; Ryan and Deci, 2000)– could be a relevant antecedent of academic PsyCap, which, in turn, –according to Conservation of Resources (COR) theory (Hobfoll, 1989)– would predict their academic performance (AP), assessed through their grade point average (GPA). In other words, academic PsyCap is expected to mediate between the satisfaction of BPN at school and AP. Providing empirical evidence about the predictive role of the school environment context for supporting BPN (Ryan and Deci, 2017b)—in academic PsyCap can help to expand our knowledge about possible ways to improve academic PsyCap through evidence–based interventions in the school community.

SDT states that there are specifiable psychological and social nutrients (the so–called BPN), which, when satisfied within the interpersonal and cultural contexts of an individual’s development, facilitate growth, integrity, and well–being (Ryan and Deci, 2017a). In other words, satisfying people’s BPN –for autonomy, competence, and relatedness– enhances their development, either in general or in specific domains, such as education (Ryan and Deci, 2000). In brief, autonomy refers to feeling willingness and volition with regard to one’s behaviors; competence refers to feeling effective in one’s interactions with the social environment; and relatedness refers to experiencing that others are responsive and sensitive as well as being able to be responsive and sensitive to others. In a school context, SDT suggests –as confirmed by previous research– that when the conditions of nurturance for holistic development are optimized (i.e., providing autonomy, competence, and relatedness), learning and educational outcomes are also optimized (Ryan and Deci, 2017b). More specifically, the satisfaction of BPN at school is associated with students’ prosocial behavior (Tian et al., 2018), gratitude (Tian et al., 2016), self–control skills (Orkibi and Ronen, 2017), academic engagement (Jang et al., 2016), and well–being (Tian et al., 2014), among others (Ratelle and Duchesne, 2014; Yu et al., 2016; Wehmeyer and Shogren, 2017).

COR theory states that individuals strive to obtain, retain, and protect their material, social, and personal resources (Hobfoll, 1989, 2002). This means that people try to accumulate resources in order to preserve and foment their health and well–being (Hobfoll, 2011). COR theory also explains –through the *caravan* notion– that resources do not occur individually, but rather they appear as co–travelers, in contrast to the general tendency in the research to focus on one resource at a time (Hobfoll et al., 2018). In this regard, the PsyCap construct is an example of a resource caravan because when the four resources it includes (i.e., hope, efficacy, resilience, and optimism) are combined into one core construct –based on their commonalities and their unique contributions–, they have a joint impact on people’s attitudes, behaviors, well–being, and performance (Luthans and Youssef–Morgan, 2017). Although initial research on PsyCap was conducted in samples of workers, more recently, the notion of academic PsyCap has received attention in both university and high school students. In these samples, direct associations are demonstrated with coping and satisfaction (Ortega-Maldonado and Salanova, 2017), academic adjustment (Liran and Miller, 2017), motivation (Siu et al., 2014), academic engagement (Carmona-Halty et al., 2019a), subjective well–being (Datu et al., 2016), and AP (Carmona-Halty et al., 2019b), among others (Vanno et al., 2014; You, 2016; Datu and Valdez, 2019).

By integrating SDT and COR theory, the present study proposes a longitudinal model that hypothesizes that academic PsyCap mediates between the satisfaction of BPN at school and AP. More specifically, we assume that when students perceive that their school environment supports their BPN during daily school life –according to SDT–, they will be more likely to have the tools they need to accumulate personal resources (in the form of academic PsyCap). In turn, these increased personal resources –according to COR theory– will lead to achieving better AP. In other words, if students’ needs for autonomy, relatedness, and competence are met at school, their hope, efficacy, resilience, and optimism about learning activities will increase, and, consequently, they will perform better. This assumption is supported, on the one hand, by research indicating that students tend to show desirable academic outcomes in situations where the social context is more supportive of their autonomy, competence, and relatedness needs (Jang et al., 2016; Orkibi and Ronen, 2017; Wehmeyer and Shogren, 2017) and, on the other hand, by research that identifies academic PsyCap as a predictor of AP (Datu et al., 2016; Ortega-Maldonado and Salanova, 2017; Carmona–Halty et al., 2018).

## Materials and Methods

### Participants

The sample was composed of 407 high school students from three Chilean educational institutions. Participants were from 12 to 18 years old (M = 14.55, SD = 1.77) and 51.4% were female. Of the 407 students, 17.2% were 12, 15.5% were 13, 17.9% were 14, 13.3% were 15, 18.7% were 16, 15.2% were 17, and 2.2% were 18 years old when the data were collected.

### Instruments

All the instruments were administered using a Spanish adaptation carried out following the guidelines of the International Test Commission for adapting tests across cultures (Muñiz et al., 2013). At time 1, the *satisfaction of BPN at school* was measured using a self–constructed scale based on the Work–related Basic Needs Satisfaction Scale (Van den Broeck et al., 2010), adapted for use in educational settings. Our scale has 12 items (e.g., “*I have the feeling that I can even accomplish the most difficult tasks at school*”) grouped into three subscales (i.e., competence, relatedness, and autonomy). All items are scored on a 7–point rating scale from 1 (*strongly disagree*) to 7 (*strongly agree*). At time 2, after 9 weeks, academic PsyCap was measured using the Academic Psychological Capital Questionnaire (Martínez et al., 2019). This questionnaire has 12 items that measure the four PsyCap components (e.g., “*I usually take stressful things in stride with regard to my studies*”) on a 6–point rating scale from 1 (*strongly disagree*) to 6 (*strongly agree*). At time 3, after another 9 weeks, AP was assessed using the grade point average (GPA) provided by the educational institutions, using four mandatory subjects in the Chilean education curriculum: mathematics, language, history, and science. According to the local grading system, GPAs ranged from 1 (*poor*) to 7 (*excellent*).

### Procedure

The recommendations of the Comité Ético-Científico (CEC–UTA) of the Chilean university host were followed in carrying out this study, and written informed consent was obtained from all subjects (i.e., the school principals, students, and students’ parents) in accordance with the Declaration of Helsinki. Participants voluntarily filled out a questionnaire on two occasions: once when the regular academic semester ended (Time 1 for the satisfaction of BPN at school) and once after a period of 9 weeks (Time 2 for academic PsyCap). In addition, AP was extracted from the teachers’ class records at the end of the next academic semester, 9 weeks later (Time 3). The same verbal and written instructions for completing the measures were provided. Participants were told to respond as truthfully as possible and assured that their responses would be anonymous. The questionnaire took about 20 min to complete using an electronic procedure.

### Data Analysis

All data were analyzed using JASP 0.9.01 and SPSS AMOS 23. For reliability analysis, Cronbach’s alpha and McDonald’s omega indexes were calculated. For structural equation modeling, we used maximum likelihood (ML) estimation methods, and goodness–of–fit was evaluated using absolute and relative indexes: Chi–square (χ^2^) and normed Chi-square (χ^2^/*df*); Incremental Fit Index (IFI); Tucker Lewis Index (TLI); Comparative Fit Index (CFI); Root Mean Square Error of approximation (RMSEA) with a 90% Confidence Interval (CI); and Standardized Root Mean Square Residual (SRMR). To determine the fit of the model, we followed the recommendations of the European Journal of Psychological Assessment (Schweizer, 2010). Finally, we implemented the bootstrapping procedure –with 5000 new samples taken from our sample (Hayes, 2009) in order to: (1) correct for any biasing impact that multivariate non–normality may have had on the computed chi–square value as a function of using ML estimation (Byrne, 2010; Kline, 2011) and (2) examine direct and indirect effects that were considered statistically significant if the 95% confidence interval estimates did not contain the value of zero.

## Results

Table 1 displays descriptive and reliability information about the study variables. It shows that internal consistencies for the scales were good and that the correlations showed significant direct relationships for all the measures used.

**TABLE 1:** Means (M), Standard Deviation (SD), Skewness (S), Kurtosis (K), Alpha (α) and Omega (ω). Reliability Indexes, and Correlations for the study variables.

	**M**	**SD**	**S**	**K**	**α**	**ω**	**1**	**2**	**3**
(1) Basic psychological needs at school	5.710	1.018	−1.068	1.360	0.790	0.798	–		
(2) Academic psychological capital	4.417	1.029	−0.595	0.187	0.910	0.912	0.612^∗∗^	–	
(3) Academic performance	5.213	0.808	−0.143	−0.668	0.886	0.888	0.295^∗∗^	0.342^∗∗^	–

*^∗∗^*p* < 0.001.*

**FIGURE 1:**
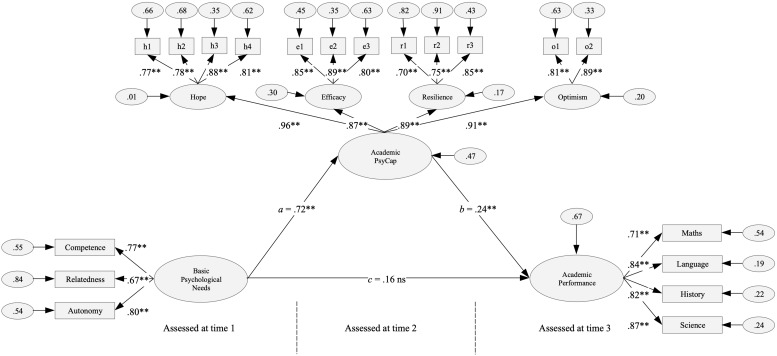
Single mediation model shows the effect of basic psychological needs on academic performance through academic PsyCap. Standardized coefficients are presented. ^∗∗^ = *p* < 0.001; ns = non-significant effect.

The proposed model contained seven latent factors and 19 indicators (see Figure 1). In other words, one factor that reflects satisfaction of BPN at school using three indicators; one higher–order factor with four lower–order factors, which, in turn, are composed of 12 indicators that make up the academic PsyCap factor; and four indicators that make up the AP factor. Results showed that this model exceeded the recommended standards and was a good representation of the data, explaining 52.0% of the academic PsyCap variance and 14.3% of the AP variance: χ^2^ = 381.965; *df* = 145; χ^2^/*df* = 2.634; IFI = 0.954; TLI = 0.946; CFI = 0.954; RMSEA = 0.063, 90% CI (0.056, 0.071); SRMR = 0.047.

Considering the good fit of the hypothesized model, direct and indirect effects were calculated and are described below. First, the satisfaction of BPN at school is significantly related to academic PsyCap [*a* = 0.721, SE = 0.042, BCa 95% CI (0.629, 0.797), *p* = 0.001]. Second, academic PsyCap is significantly related to AP, after controlling for the satisfaction of BPN at school [*b* = 0.241, SE = 0.087, BCa 95% CI (0.071, 0.405), *p* = 0.010]. Third, the satisfaction of BPN at school is significantly and indirectly associated with AP through academic PsyCap [*ab* = 0.174, SE = 0.063, BCa 95% CI (0.056, 0.300), *p* = 0.008]. In addition, because the satisfaction of BPN at school is not significantly related to AP [*c* = 0.165, SE = 0.098, BCa 95% CI (−0.028, 0.356), *p* = 0.088], we can conclude that academic PsyCap fully mediates the relationship between the satisfaction of BPN at school and AP.

## Discussion

The current paper makes several theoretical contributions. First, we found that the satisfaction of students’ BPN is directly related to their academic PsyCap. Second, we found that the accumulation of personal resources in the form of academic PsyCap is directly related to students’ AP. Third, we found that students whose BPN is satisfied at school (i.e., competence, relatedness, and autonomy) are more likely to achieve better academic results (i.e., GPA score) through academic PsyCap (i.e., a resource caravan formed by hope, efficacy, resilience, and optimism). Taken together, these results are in line with previous research on SDT and COR theory and they make an innovative contribution to the scarce empirical research on school environment variables as antecedents of academic PsyCap (e.g., Orkibi and Ronen, 2017; Wehmeyer and Shogren, 2017; Carmona-Halty et al., 2019b; Datu and Valdez, 2019).

As a main practical implication, we want to emphasize the key role that educational institutions (can) play in increasing students’ academic PsyCap. That is, instead of focusing on a curriculum based on control and achievement –which thwarts students’ BPN fulfillment– the school community should concentrate on creating an environment that sets clear rules and gives students positive feedback (i.e., supporting the need for competence), expresses interest and care for them (i.e., supporting the need for relatedness), and provides them with the freedom to make their own choices (i.e., supporting the need for autonomy). This proposal is coherent with the *resource caravan passageways* notion, which points out that people’s resources exist in an environment that either fosters/nurtures or limits/blocks resource creation or nourishment (see Hobfoll, 2011; Hobfoll et al., 2018). Therefore, the PsyCap components should be enhanced not only through individual-level interventions— using the Psychological Capital Intervention method (Luthans et al., 2008, 2010)—, but also through school-level promotion, including a supportive educational curriculum as a strategy to obtain –based on our results– better academic performance.

This study has several strengths. First, we used a longitudinal three–wave design, which agrees with the temporal sequence that we assumed in the proposed mediational model. Second, we included the GPA as an objective academic performance indicator. Third, we empirically demonstrated the integration of both SDT and COR theory in an academic setting. However, this study has also some weaknesses. First, we used a convenience sample instead of a representative Chilean student sample. Second, we used self–reports for both psychological measures instead of using, for instance, peer or teacher ratings. Third, we focused on unidirectional effects instead of examining bi–directional effects. Fourth, our three assessment points (i.e., BPN, PsyCap, and AP) only cover short–term effects (approximately 5 months) instead of capturing long–term effects (e.g., using a 2 year longitudinal design). Finally, for future research, we would suggest the following. First, it would be interesting to examine an alternative model proposing that students with high satisfaction of BPN and high PsyCap would have higher AP, whereas students with low satisfaction of needs and low PsyCap would have lower AP. Second, based on the recent team–level PsyCap approach, the applicability of class–level PsyCap should be explored in order to examine its role in students’ individual and/or class–level outcomes such as academic engagement, academic burnout, and AP. Third, the inclusion of other school environment variables –such as a sense of community– in a more comprehensive model would be a fruitful future research agenda to expand our knowledge about academic PsyCap antecedents.

## Data Availability

The datasets generated for this study are available on request to the corresponding author.

## Ethics Statement

The studies involving human participants were reviewed and approved by Comité Ético-Científico (CEC-UTA). Written informed consent to participate in this study was provided by the participants’ legal guardian/next of kin.

## Author Contributions

All authors listed have made a substantial, direct and intellectual contribution to the work, and approved it for publication.

## Conflict of Interest Statement

The authors declare that the research was conducted in the absence of any commercial or financial relationships that could be construed as a potential conflict of interest.
